# Lanifibranor Reduces Inflammation and Improves Dyslipidemia in Lysosomal Acid Lipase-Deficient Mice

**DOI:** 10.1016/j.gastha.2024.05.006

**Published:** 2024-05-28

**Authors:** Ivan Bradić, Nemanja Vujić, Katharina B. Kuentzel, Hansjörg Habisch, Anita Pirchheim, Alena Akhmetshina, John D. Henderson, Tobias Madl, Atul S. Deshmukh, Dagmar Kratky

**Affiliations:** 1 Division of Molecular Biology and Biochemistry, Gottfried Schatz Research Center, Medical University of Graz, Graz, Austria; 2 Novo Nordisk Foundation Center for Basic Metabolic Research, University of Copenhagen, Copenhagen, Denmark; 3 BioTechMed-Graz, Graz, Austria

**Keywords:** LAL, Lysosomal Acid Lipase Deficiency, Liver Inflammation, Lipoproteins, Pan-PPAR Agonist

## Abstract

**Background and Aims:**

Recent studies showed that patients suffering from lysosomal acid lipase deficiency (LAL-D) benefit from enzyme replacement therapy; however, liver histopathology improved in some but not all patients. We hypothesized that the pan-peroxisome proliferator-activated receptor agonist lanifibranor may have beneficial effects on liver inflammation in LAL knockout (Lal−/−) mice based on its promising results in alleviating liver inflammation in patients with metabolic dysfunction-associated steatohepatitis.

**Methods:**

Female Lal−/− mice were daily gavaged with lanifibranor or vehicle for 21 days. The effects of the treatment were assessed by measuring body and organ weights, plasma lipids and lipoproteins, as well as hematological parameters, followed by liver proteomics and metabolomics.

**Results:**

Lanifibranor treatment slightly altered organ weights without affecting the total body weight of Lal−/− mice. We observed major changes in the proteome, with multiple proteins related to lipid metabolism, peroxisomal, and mitochondrial activities being upregulated and inflammation-related proteins being downregulated in the livers of treated mice. Hepatic lipid levels and histology remained unaltered, whereas plasma triacylglycerol and total cholesterol levels were decreased and the lipoprotein profile of lanifibranor-treated Lal−/− mice improved.

**Conclusion:**

Lanifibranor treatment positively affected liver inflammation and dyslipidemia in Lal−/− mice. These findings suggest the necessity of a further combined study of lanifibranor with enzyme replacement therapy in Lal−/− mice to improve the phenotype. Moreover, there is a compelling rationale for conducting clinical trials to assess the efficacy of lanifibranor as a potential treatment option for LAL-D in humans.

## Graphical abstract

**Figure:**
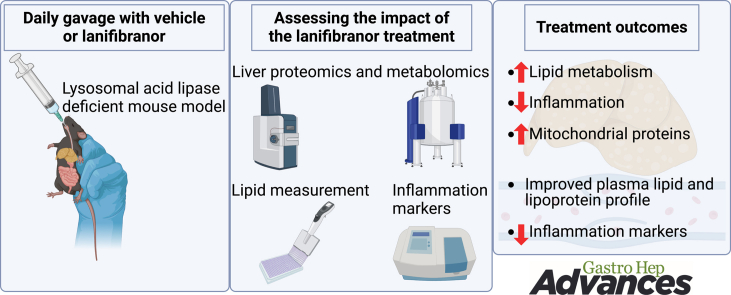

## Introduction

Lysosomal acid lipase deficiency (LAL-D) is a lysosomal storage disorder that affects 1 in 177,452 individuals.^1^ It may masquerade as metabolic dysfunction-associated steatotic liver disease (MASLD) or present with cryptogenic cirrhosis.^1^ LAL activity is reduced in adult MASLD^2^^,^^3^ and even more so in metabolic dysfunction-associated steatohepatitis (MASH) and cryptogenic cirrhosis.^4^^,^^5^ Furthermore, a correlation was found between decreased LAL activity and the extent of liver fibrosis in pediatric patients with MASLD.^6^ MASLD is associated with the accumulation of high levels of fatty acids, triacylglycerols (TG), and ceramides in the liver.^7^ Depending on the mutations in the *LIPA* gene and the residual LAL activity, patients develop very severe (early-onset) or less severe (late-onset) LAL-D.^8^ Since LAL is the sole enzyme known to degrade cholesteryl esters and TG in the acidic lysosomal lumen, a lack of the enzymatic activity causes lysosomal accumulation of the substrates.^8^ In patients suffering from early-onset LAL-D, the massive ectopic lipid deposition affects multiple organs, leading to death within the first few months of life due to malabsorption, cachexia, and failure to thrive.^9^ In contrast, patients with late-onset LAL-D survive into adulthood and usually present with hepatosplenomegaly, dyslipidemia, and atherosclerosis.^9^

Similar to humans, LAL knockout (Lal−/−) mice exhibit lipodystrophy, ectopic lipid accumulation, progressive hepatosplenomegaly, and dyslipidemia.^10^, ^11^, ^12^ The phenotype of Lal−/− mice resembles late-onset LAL-D, despite having no residual LAL activity.^10^, ^11^, ^12^ Lal−/− mice were used to test various treatment approaches for LAL-D, including enzyme replacement therapy (ERT) and gene therapy.^13^^,^^14^ Particularly, ERT with recombinant human LAL showed promising preclinical outcomes by reducing the size of the liver and spleen and decreasing lipid accumulation in multiple tissues of Lal−/− mice.^13^ Currently, human LAL ERT is the most effective therapeutic strategy for LAL-D patients,^8^ leading to an improvement of liver injury markers and dyslipidemia.^15^ However, mild to moderate adverse effects were reported in the majority of treated patients.^8^ In addition, the impact of ERT on liver histology and inflammation is inconclusive,^16^ as some patients exhibited improvements, while others experienced a worsening of the liver phenotype.^15^ Disease severity and its specific stage, LAL-D-dependent cumulative liver injury and age, development of neutralizing antibodies against the drug, or other epigenetic factors may influence the efficacy of ERT treatment in LAL-D patients.

We recently showed that the livers of Lal−/− mice have impaired lysosomal function and decreased expression of proteins related to fatty acid metabolism and peroxisomes.^17^ In addition, genetic loss of LAL and pharmacological inhibition of lysosomal function^18^ downregulate peroxisome proliferator-activated receptor (PPAR) α signaling and decrease peroxisomal biogenesis and lipid degradation. PPARs are a family of nuclear receptors that regulate whole-body energy metabolism, inflammation, peroxisomal biogenesis, and fatty acid metabolism.^19^^,^^20^ Due to their distinct functions, PPARα is primarily expressed in the liver, PPARγ in adipocytes, and PPARδ is expressed ubiquitously. Single and dual PPAR agonists have shown promising results in the treatment of diabetes and MASLD-related complications.^21^ The pan-PPAR agonist lanifibranor combines the benefits of selective PPAR agonists by targeting all 3 PPAR isoforms and successfully reduces hepatic steatosis, inflammation, and fibrosis in MASLD mouse models.^22^ Treatment with lanifibranor also improved liver inflammation and might lead to regression of fibrosis in MASH patients, as evidenced by the positive impact of lanifibranor on resolution of MASH without worsening of fibrosis and the regression of fibrosis without a deterioration of MASH from the phase 2b trial.^23^ The lanifibranor-treated group exhibited a dropout rate of less than 5% due to adverse events, which were primarily mild or moderate in intensity. The efficacy and safety of lanifibranor in the treatment of MASH with fibrosis are currently being tested in a large multicenter phase 3 clinical trial (NCT04849728, EudraCT Number: 2020-004986-38).

The aim of the study was to investigate the potential of lanifibranor to ameliorate liver inflammation in Lal−/− mice. The results demonstrate that pan-PPAR agonism, even without ERT, significantly improved dyslipidemia and hepatic inflammation without negatively affecting liver lipid parameters in Lal−/− mice. These findings suggest that targeting PPAR signaling may be an effective approach to alleviating hepatic inflammation in LAL-D and warrant further studies to establish the therapeutic potential of lanifibranor.

## Methods

### Animals and Pharmacological Treatment

The mice were housed in a clean and temperature-controlled (22 ± 1 °C) environment and had unlimited access to chow diet (Altromin 1324, Lage, Germany) and water on a regular 12-hour light/12-hour dark cycle. The experiments were performed using 10 female Lal−/− mice aged 7–11 weeks on the C57BL/6J background,^12^ which were randomly selected. Six mice were gavaged a suspension of 1% methylcellulose and 0.1% poloxamer in water (vehicle), while 4 mice were gavaged with the vehicle suspension supplemented with 30 mg/kg of lanifibranor once daily for 21 days, which has already been described to exhibit antifibrotic activity in a liver fibrosis model.^24^ All treated mice were sacrificed 24 hours after the final treatment and fasted for 4 hours prior to sacrifice.

The animal experiments were conducted in accordance with the European Directive 2010/63/EU, complying with national laws, and approved by the Austrian Federal Ministry of Education, Science, and Research, Vienna, Austria (2022-0.920.281, BMWFW-66.010/0109-WF/V/3b/2015, 2022-0.861.148).

### Plasma Lipid and Lipoprotein Quantification

Plasma TG and cholesterol concentrations were determined from ethylenediaminetetraacetic acid blood as previously described.^17^ Lipoprotein profiles were obtained after the separation of 200 μL pooled plasma by fast protein liquid chromatography (Pharmacia P-500, Uppsala, Sweden) equipped with a Superose 6 column (Amersham Biosciences, Piscataway, NJ).

### Tissue Lipid Extraction and Quantification

Liver tissue was lysed in phosphate-buffered saline (PBS) using a Precellys homogenizer (Bertin Instruments, Bretonneux, France) and then centrifuged at 8000 × *g* for 2 minutes at 4 °C. From the supernatant, an equivalent of 20 mg of tissue was transferred into a new tube, and the volume was filled up to 100 μL with PBS. The samples were treated with 750 μL of methanol and 2.5 mL of methyl tert-butyl ether, vortexed for 10 seconds, and rotated for 1 hour. After the addition of 600 μL ddH_2_O, the samples were vortexed for 10 seconds and left for 10 minutes. The phases were separated by centrifugation at 2000 × *g* for 10 minutes. The upper organic phase was collected, to which 200 μL of 2% TritonX-100 in methanol was added before evaporation under a N_2_ stream. Two hundred microliters of ddH_2_O were added to the samples, and the precipitates were dissolved by vortexing for 20 seconds, followed by incubation in a sonication bath for 1 hour and with a tip-sonicator for 10 seconds. The lipid levels were determined as described above.

### Hematoxylin and Eosin and Masson's Trichrome Staining

Fresh liver tissue was fixed in 4% PBS-buffered paraformaldehyde for 24 hours and embedded in paraffin. Slides with deparaffinized tissue sections were incubated with hematoxylin for 10 minutes and then with eosin for 1 minute. Masson's trichrome staining was performed as previously described.^25^

### Measurement of Liver Injury Marker

Freshly isolated heparinized blood was centrifuged for 7 minutes at 5200 × *g* and 4 °C, and plasma was collected. Alanine aminotransferase and aspartate aminotransferase (AST) concentrations in plasma were analyzed with Fuji Dri-Chem NX500 (FUJIFILM Holdings Corporation, Tokyo, Japan).

### Measurement of Hematological Parameters and Blood Glucose

Blood samples were collected in ethylenediaminetetraacetic acid-coated tubes and analyzed using the V-Sight Vet Hematology Analyzer (A. Menarini Diagnostics, Florence, Italy) according to the manufacturer's protocol.

Glucose was measured from a drop of tail vein blood using a glucometer (AccuCheck, Roche Holding AG, Basel, Switzerland).

### Sample Preparation for Mass Spectrometry-Based Proteomics and Measurement

One milliliter of sodium dodecyl sulfate buffer (0.1 M Tris-HCl, pH 8.5%, and 4% sodium dodecyl sulfate) was added to approximately 100 mg liver tissue from Lal−/− mice. Samples were homogenized in a BeatBox tissue homogenizer (PreOmics GmbH; Martinsried, Germany) at 850 rpm and boiled for 10 minutes at 95 °C. Lysates were sonicated using a tip-sonicator and centrifuged at 16,000 × *g* for 10 minutes. Protein concentrations were determined in the supernatant using the DC Protein Assay (Bio-Rad Laboratories, Hercules, CA). Proteins were reduced and alkylated with final concentrations of 10 mM tris(2-carboxyethyl)phosphine and 50 mM 2-chloroacetamide. Proteins were digested using the protein aggregation capture method^26^ on a KingFisher Flex robot (Thermo Fisher Scientific, Waltham, MA) in 96-well format. Briefly, magnetic hydroxyl beads (ReSyn Biosciences, Gauteng, South Africa) were mixed with 30 μg protein lysate 1:4 (w:w) in the 96-well plate. A digestion solution containing endoproteinases LysC (1:500, w:w) and trypsin (1:100, w:w) was added to the second 96-well plate. Five additional 96-well plates containing 500 μL 100% acetonitrile (ACN), 700 μL 100% ACN, 1 mL 100% ACN, 1 mL 100% ethanol, and 1 mL 100% ethanol, respectively, were prepared. An in-house program was used to digest the samples. Peptides were purified with 3 × C18 StageTips.^27^ EvoTips (Evosep, Odense, Denmark) were loaded with 200 ng of purified peptides, which were then separated on a 15-cm column with 150 μM ID packed with C18 beads (1.9 μm) (Pepsep) using Evosep ONE HPLC. The separated peptides were injected into a timsTOF Pro 2 mass spectrometer (Bruker Daltonics, Bremen, Germany) operated in diaPASEF mode, utilizing a CaptiveSpray ionization source with a 20-μm emitter. Mass spectrometry (MS) data were collected between 100 and 1700 m/z. Each MS/MS data acquisition was performed with diaPASEF cycle of 1.8 s and covered ion mobility range between 1.6 and 0.6 1/K_0_. Ion mobility was calibrated with 622.0289, 922.0097, and 1221.9906 Agilent ESI-L Tuning Mix ions. For diaPASEF, a long gradient with 10 diaPASEF scans with 3 25 Da windows per ramp, a mass range between 400 and 1201 Da, and a mobility range between 1.43 and 0.6 1/K_0_ was used. The collision energy parameters were set to 59 eV at 1/K_0_ = 1.3 and linearly decreased to 20 eV at 1/K_0_ = 0.85 Vs cm^−2^ during the measurement. Accumulation time and PASEF ramp time were 100 ms.

### Proteomics Data Quantification and Bioinformatics

Protein quantification was performed with DIA-NN (1.8.1),^28^^,^^29^ with raw data searched against the Mouse Uniprot reviewed FASTA file downloaded on August 09, 2021 with the following parameters: FASTA digest for library-free search/library generation, deep learning-based spectra, retention time, and ion mobility spectra prediction. Trypsin-digested peptides with up to 2 missed cleavages were accepted, along with 3 variable modifications: N-terminal methionine excision, cysteine carbamidomethylation, and methionine oxidation. The default peptide parameters were 7–30 for the peptide length range, 1–4 for the precursor charge range, 300–1800 for the precursor m/z range, and 200–1800 for the fragment ion m/z range. The precursor false discovery rate (FDR) was set to 1%, mass accuracy to 10 ppm, and MS1 accuracy to 20 ppm. Isotopologues were used in the search, along with match between runs, heuristic protein inference (protein inference was set to genes), and exclusion of shared spectra. Searches were conducted in double-pass mode using a robust, high-precision liquid chromatography quantification strategy, with cross-run normalization as a function of retention time and smart profiling for library generation. Data were analyzed using Perseus (1.6.15.0)^30^ and Jupyter Notebook with Python 3.9. Protein intensities were log_2_ transformed before filtering the data for 70% of valid values in at least 1 group. Data were imputed to fill missing abundance values by drawing random numbers from a Gaussian distribution with a standard deviation of 30% and a downshift of 1.8 standard deviations from the mean relative to that of the proteome abundance distribution. Principal component analysis (PCA) was performed on the z-scored data in Python utilizing the packages Pandas, Matplotlib, Numpy, Seaborn, Sklearn, and Bioinfokit. Statistically significant changes were determined by a *t*-test with S0 = 0.1 and FDR = 0.05. Reactome pathway enrichment with the PANTHER Overrepresentation Test^29^^,^^30^ was performed using 2 clusters representing significantly increased proteins in the livers of lanifibranor- or vehicle-treated Lal−/− mice. KEGG pathway enrichment was performed with z-scored intensities from significantly changed proteins using Pathview.^31^^,^^32^ PPAR signaling was selected from enriched KEGG pathways and changed proteins related to it were counted and visualized. The number of significantly upregulated proteins from livers of vehicle- and lanifibranor-treated mice associated with specific UniProt keywords was determined by counting annotated proteins (Swiss-Prot) from relevant UniProt keywords.

Protein matrix, enriched Reactome pathways, and proteins related to specific UniProt keywords are listed in Table A1.

### Nuclear Magnetic Resonance (NMR) Metabolomics

Metabolomics analysis by NMR spectroscopy was performed as previously described.^33^ Briefly, 20 mg of liver tissue was mixed with a solution of ice-cold methanol and high-purity H_2_O (2:1) to inhibit enzymatic processes and precipitate proteins. Samples were homogenized 2 times for 20 seconds using a Precellys 24 tissue homogenizer (Bertin Technologies, Montigny-le-Bretonneux, France) in tubes containing Precellys beads (1.4 mm zirconium oxide beads, Bertin Technologies). Next, samples were centrifuged at 9703 × *g* for 30 minutes at 4 °C, the supernatants were transferred to new tubes and lyophilized at <1 Torr, 824 × *g*, 25 °C for 10 hours in a vacuum-drying chamber (Savant Speedvac SPD210 vacuum concentrator) with an attached cooling trap (Savant RVT450 refrigerated vapor trap) and vacuum pump (VLP120) (Thermo Scientific, Waltham, MA). Samples were redissolved in 500 μL NMR buffer consisting of D_2_O, 0.08 M Na_2_HPO_4_, 4.6 mM 3-(trimethylsilyl) propionic acid-2,2,3,3-d4 sodium salt (TSP), and 0.04 (w/v)% NaN_3_ (pH adjusted to 7.4 with HCl and NaOH) and transferred to 5-mm NMR tubes. NMR spectroscopy was carried out using a Bruker Avance Neo 600 MHz spectrometer coupled to a TXI probe head at 310 K. ^1^H 1D NMR spectra were obtained using the Carr–Purcell–Meiboom–Gill (CPMG) pulse sequence (cpmgpr1d, 128 scans, 73,728 points in F1, 12,019.230 Hz spectral width, recycle delay 4 seconds) with a pre-saturation for water suppression. NMR spectra processing, including Fourier transformation of the free induction decay, automatic phasing, and baseline correction, was done using Bruker Topspin software version 4.0.2. A Matlab script (software version 2014b) was used for importing the spectra. The peaks surrounding the water, TSP, and methanol signals were eliminated, the NMR spectra were aligned,^34^ and a probabilistic quotient normalization^35^ was carried out. Chenomx NMR Suite 8.4 (Chenomx Inc, Edmonton, AB, Canada), the human metabolome database,^36^ and reference chemicals were used to identify metabolites. Metabolites were quantified by signal integration of the normalized spectra. For each metabolite, a representative peak with no overlapping signals was determined. The start and end points of the integration were then selected to revolve around this peak, and the areas of all peaks were integrated for all samples and metabolites using an R script (RStudio 2023 with R version 4.1.3) and provided as arbitrary units (a.u.) proportional to the concentration of the respective analyte. MetaboAnalyst 5.0^37^ was used to calculate univariate statistics (shown as volcano plot). An unpaired 2-tailed Student's *t*-test with Benjamini-Hochberg correction was used to calculate the significance of metabolite levels between controls and lanifibranor-treated mice. Since none of the metabolites revealed an FDR <0.05, uncorrected *P* values were plotted. For multivariate statistics, PCA and orthogonal partial least squares discriminant analysis were calculated. The statistical significance of the multivariate model was verified by permutation testing of the quality evaluation statistic Q^2^, which was barely significant (*P* = .11).

### Statistics

Data analysis and statistics for proteomics and metabolomics data are described in the sections above. For other experiments, comparisons between groups were performed using the unpaired 2-tailed Student's *t*-test in GraphPad Prism 9.5.1. Data are presented as mean or as mean ± SD. Significance levels were set to: ∗*P* < .05, ∗∗*P* ≤ .01, ∗∗∗*P* ≤ .001, and ∗∗∗∗*P* ≤ .0001.

## Results

### Lanifibranor Treatment Causes Minor Changes in Organ Weight but Not Body Weight in Lal−/− Mice

**Figure 1:**
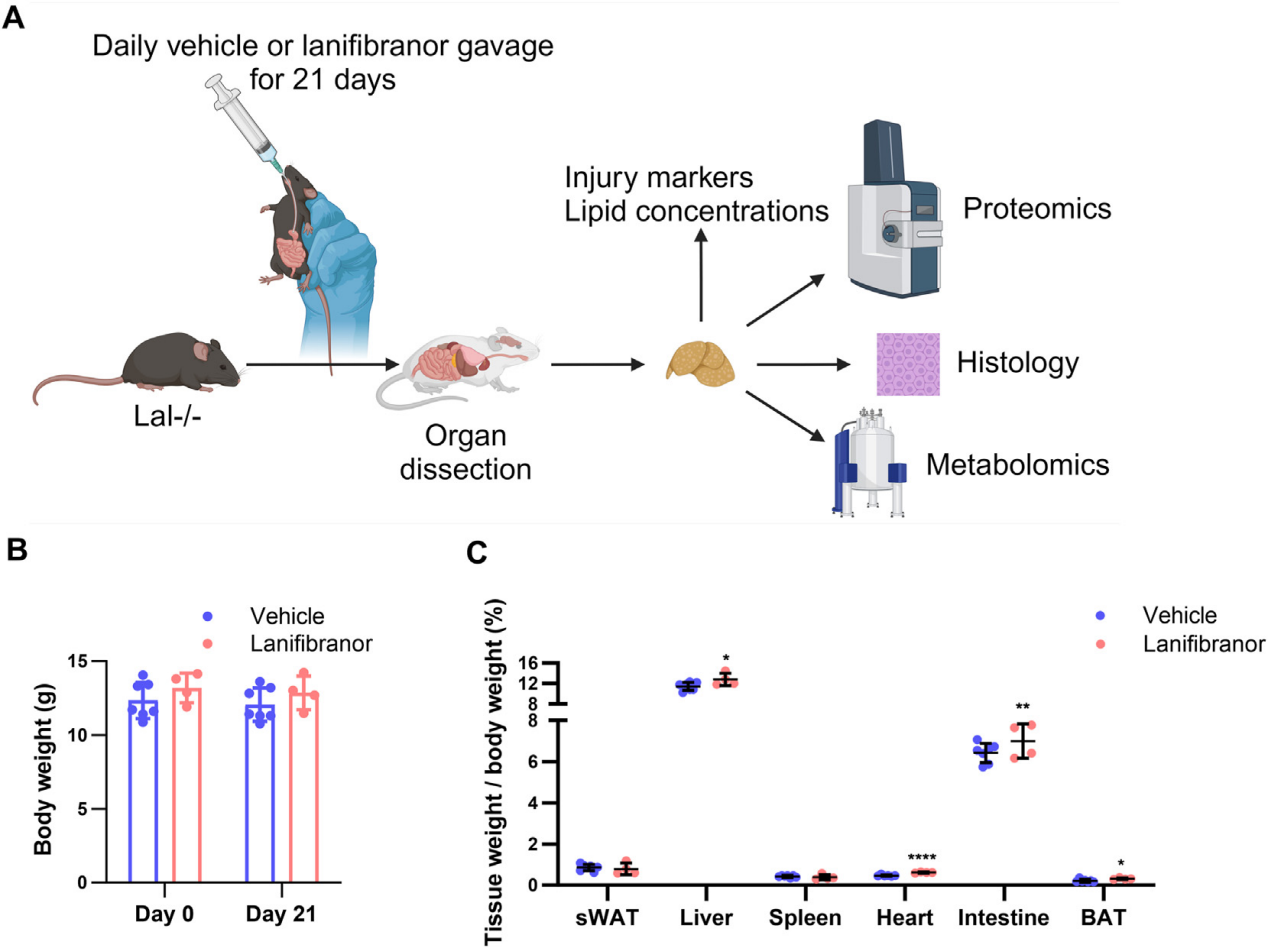
Lanifibranor treatment causes minor changes in organ weight but not body weight in lysosomal acid lipase (LAL) knockout (Lal−/−) mice. (A) Lal−/− mice were daily gavaged with a suspension of methylcellulose (1%) and poloxamer (0.1%) in water (vehicle) with or without 30 mg/kg lanifibranor for 21 days (created with BioRender.com). (B) Body weight and (C) ratio of tissue weight to body weight of lanifibranor- or vehicle-treated Lal−/− mice. Data represent mean values ± SD (n = 4–6), and statistical significance was determined by 2-tailed Student's *t*-tests. ∗*P* < .05, ∗∗*P* ≤ .01, ∗∗∗∗*P* ≤ .0001. BAT, brown adipose tissue; sWAT, subcutaneous white adipose tissue.

**Figure A1:**
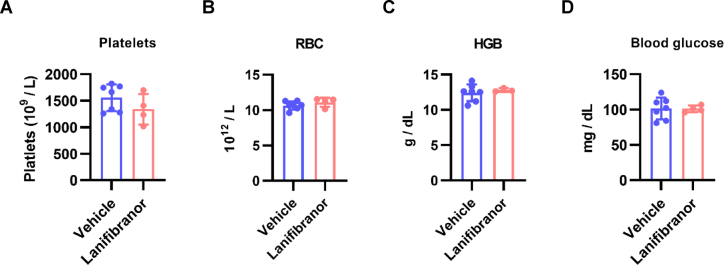
Unchanged hematological parameters and blood glucose levels after lanifibranor treatment in Lal−/− mice. Values of (A) platelets (PLT), (B) red blood cells (RBC), (C) hemoglobin (HGB), (D) blood glucose in vehicle- or lanifibranor-treated Lal−/− mice. Data represent n = 4–6.

We assessed the effects of lanifibranor treatment on the phenotype of female Lal−/− mice after 21 days of daily gavage with vehicle or lanifibranor (Figure 1A). The body weight of both groups was comparable (Figure 1B). The tissue-to-body weight ratio of liver, heart, small intestine, and brown adipose tissue was increased in lanifibranor-treated mice by 1.12, 1.32, 1.21, and 1.49 fold, respectively, whereas the ratio of subcutaneous white adipose tissue and spleen remained unchanged (Figure 1C). We next measured several hematologic parameters to determine the functional consequences of increased liver and heart weight in lanifibranor-treated mice but observed no changes in platelet number (Figure A1A), red blood cell number (Figure A1B), and hemoglobin levels (Figure A1C).

### Lanifibranor Treatment Activated the PPAR Signaling Pathway in the Livers of Lal−/− Mice

**Figure 2:**
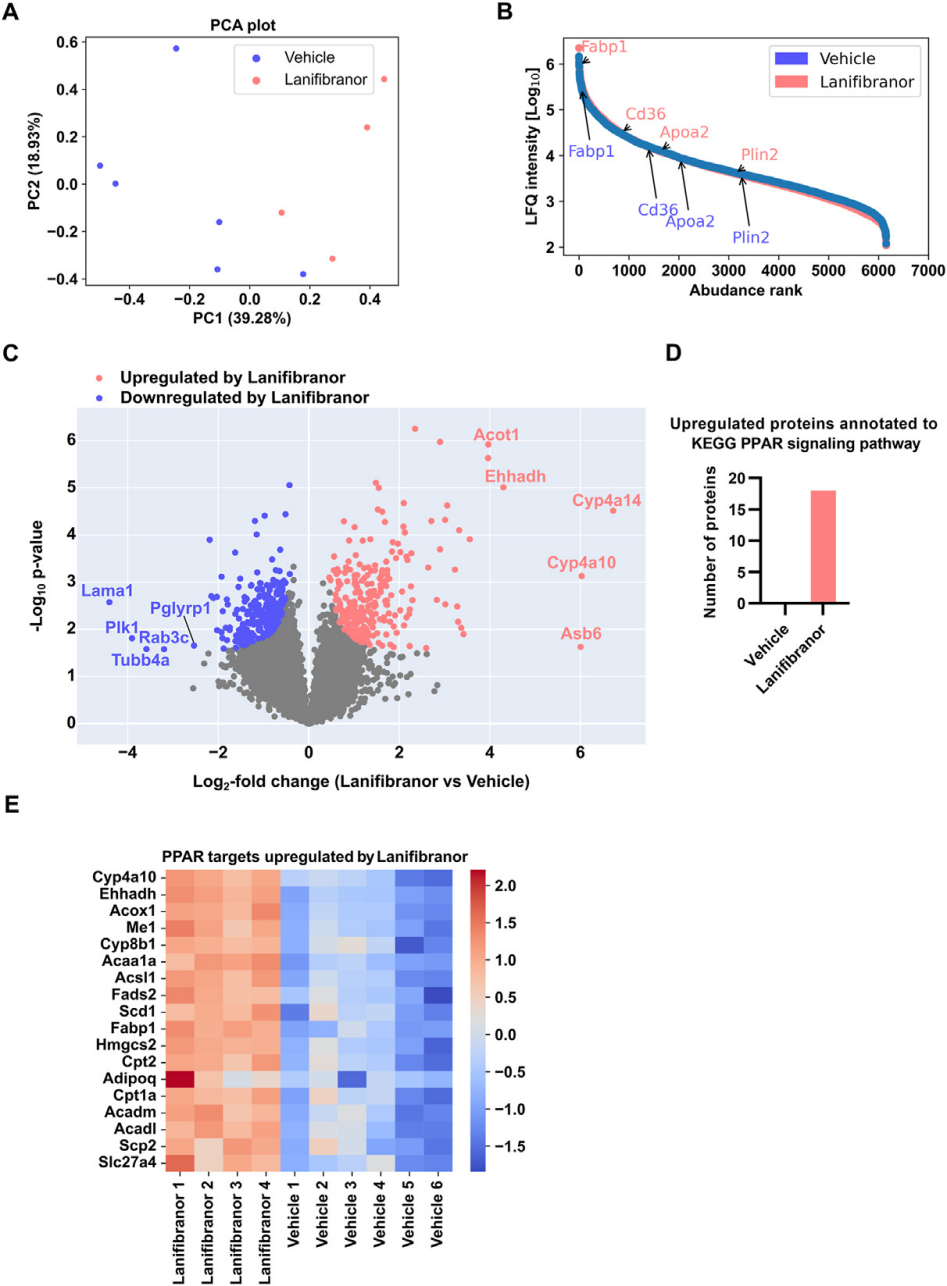
Lanifibranor increased the expression of peroxisome proliferator-activated receptor (PPAR) signaling-associated proteins. (A) Plot of principal component analysis (PCA) and (B) the dynamic range of the quantified liver proteome with some PPAR target proteins upregulated in the livers of lanifibranor-treated Lal−/− mice. (C) The volcano plot displays 254 significantly increased and 246 significantly decreased proteins in the livers of lanifibranor-treated Lal−/− mice. (D) The number of significantly changed proteins associated with the KEGG PPAR signaling pathway and (E) their heatmap representing all significantly altered proteins annotated in the livers of vehicle- or lanifibranor-treated Lal−/− mice (n = 4–6). Statistical significance was determined by 2-tailed Student's *t*-tests with permutation-based FDR (FDR < 0.05, S0 = 0.1).

To identify potential changes in protein expression following lanifibranor treatment, we performed untargeted label-free quantitative proteomic analysis on livers of lanifibranor- and vehicle-treated Lal−/− mice. The distinct clustering pattern of the data by PCA indicated differences between the 2 groups, with principal component 1 (PC1) explaining 39.3% and PC2 explaining 18.9% of the variance (Figure 2A). The abundance of 6151 quantified proteins covered 4 orders of magnitude, and the detection of multiple PPAR target proteins (Figure 2B) demonstrated the good quality of the proteomics data. Of the 510 significantly changed proteins, 256 were upregulated and 254 were downregulated in the livers of lanifibranor-treated Lal−/− mice (Figure 2C), indicating substantial remodeling of the proteome following treatment. The highest upregulated proteins included acyl-coenzyme A thioesterase 1 (ACOT1), peroxisomal bifunctional enzyme (EHHADH), cytochrome P450 4A14 (CYP4A14), CYP4A10, and ankyrin repeat and SOCS box containing 6 (ASB6), whereas peptidoglycan recognition protein 1 (PGLYRP1), ras-related protein Rab-3C (RAB3C), tubulin beta 4A class IVa (TUBB4A), serine/threonine-protein kinase PLK1 (PLK1), and laminin subunit alpha 1 (LAMA1) were among the 5 most downregulated proteins (Figure 2C). Of the 43 proteins annotated to the KEGG PPAR signaling pathway, 18 were upregulated in the livers of lanifibranor-treated mice, whereas none was upregulated in control livers (Figure 2D). The z-scored intensities of the upregulated proteins involved in PPAR signaling clearly demonstrated that treatment with the pan-PPAR agonist lanifibranor successfully triggered the expression of multiple PPAR targets, with the most pronounced effects on CYP4A10, EHHADH, and peroxisomal acyl-coenzyme A oxidase 1 (ACOX1) (Figure 2E).

### Functional Interpretation of Activated PPAR Signaling

**Figure 3:**
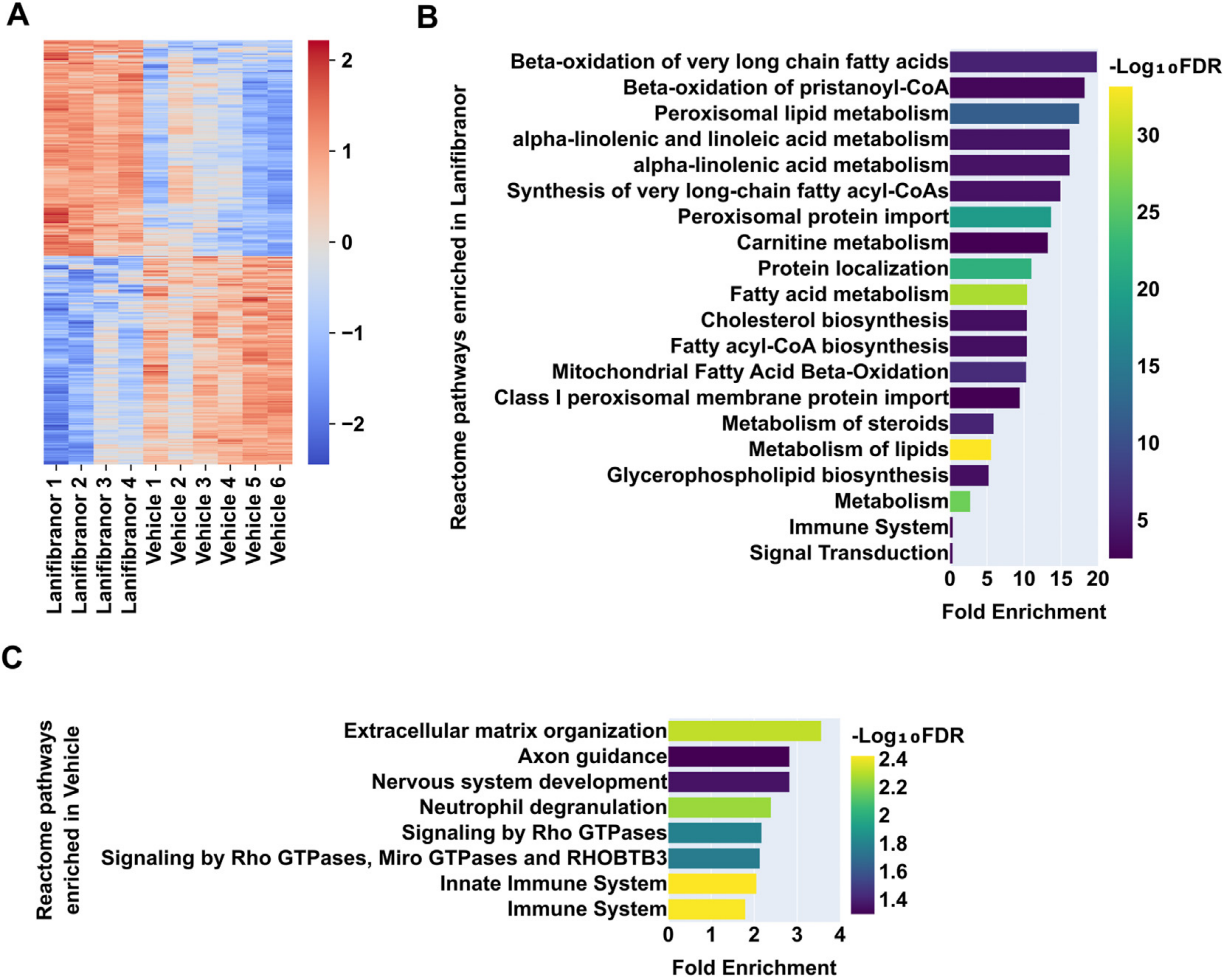
Treatment with lanifibranor induces peroxisome and lipid metabolism-related pathways in the livers of Lal−/− mice. (A) Heatmap of z-scored significantly changed proteins in the livers of lanifibranor- and vehicle-treated Lal−/− mice. (B) The highest significantly enriched Reactome pathways of proteins significantly upregulated and (C) downregulated in the livers of lanifibranor-treated Lal−/− mice. Data represent n = 4–6. Statistical significance was determined by (A) 2-tailed Student's *t*-tests with permutation-based FDR (FDR < 0.05, S0 = 0.1) and (B and C) by Fisher's exact with Benjamini-Hochberg correction (FDR < 0.05).

To functionally interpret the lanifibranor-induced proteome changes, we performed an overrepresentation analysis on 2 clusters representing significantly upregulated and downregulated proteins in livers of lanifibranor-treated Lal−/− mice (Figure 3A). Numerous Reactome pathways were enriched, including fatty acid beta-oxidation, peroxisomal lipid metabolism, peroxisomal protein import, cholesterol biosynthesis, and lipid metabolism (Figure 3B). In contrast, immune system and signal transduction were among the pathways that were less enriched, indicating decreased inflammation in the livers of lanifibranor-treated mice (Figure 3B). Consistent with this finding, several inflammation-related Reactome pathways, including neutrophil degranulation and (innate) immune system, were enriched in control livers (Figure 3C). Extracellular matrix organization was the most enriched Reactome pathway in control livers, suggesting a profound impact of lanifibranor on extracellular matrix remodeling (Figure 3C). These data indicated that lanifibranor stimulated various processes related to lipid metabolism and peroxisomes and exhibited a favorable impact on liver inflammation in Lal−/− mice.

### Lanifibranor Treatment Affects the Liver Proteome of Peroxisomes, Mitochondria, Lipid Metabolism, and Extracellular Matrix Proteins

**Figure 4:**
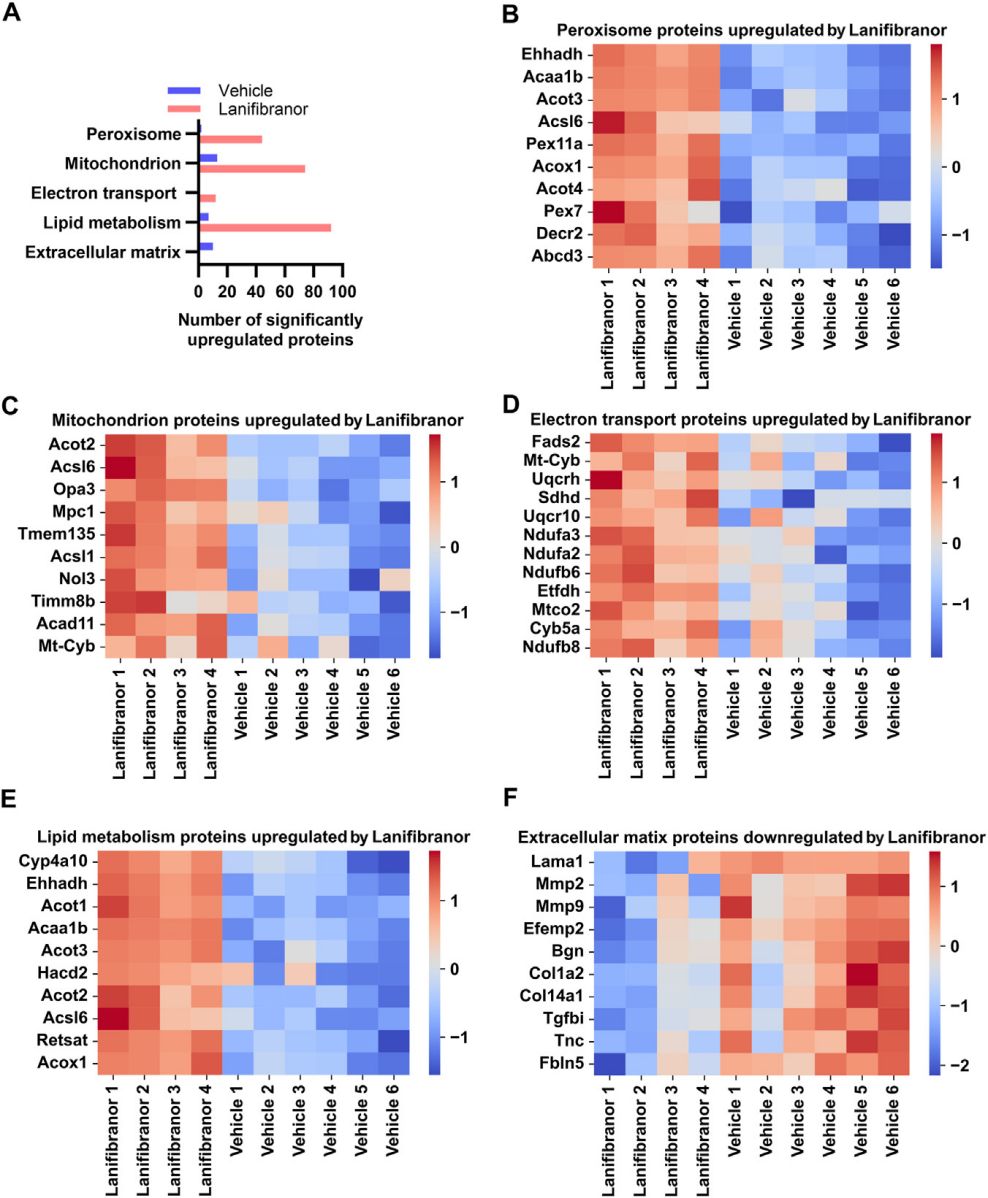
Lanifibranor treatment triggers the expression of proteins associated with peroxisomes, mitochondria, and lipid metabolism in the livers of Lal−/− mice. (A) The number of significantly changed proteins annotated to specific UniProt keywords in the livers of vehicle- or lanifibranor-treated Lal−/− mice. Heatmaps representing the most upregulated proteins in the livers of lanifibranor-treated Lal−/− mice annotated to (B) peroxisome, (C) mitochondrion, (D) electron transport, and (E) lipid metabolism. (F) Heatmap showing the most downregulated proteins annotated to the extracellular matrix. Data represent n = 4–6. Statistical significance was determined by 2-tailed Student's *t*-tests with permutation-based FDR (FDR < 0.05, S0 = 0.1).

To further scrutinize the proteomic changes induced by lanifibranor treatment, we quantitated the number of significantly dysregulated proteins annotated to specific UniProt keywords in the livers of lanifibranor- and vehicle-treated Lal−/− mice. Administration of lanifibranor triggered the upregulation of 44 proteins associated with peroxisomes, 74 with mitochondria, 12 with electron transport, and 92 with lipid metabolism (Figure 4A). In contrast, livers of control mice had only 2, 13, 0, and 7 significantly upregulated proteins associated with peroxisomes, mitochondria, electron transport, and lipid metabolism, respectively (Figure 4A). Increased abundance of 3-ketoacyl-CoA thiolase B, peroxisomal (ACAA1B), ACOT4, long-chain fatty acid CoA ligase 6 (ACSL6), peroxisomal membrane protein 11A (PEX11A), and peroxisomal acyl-CoA oxidase 1 (ACOX1) indicated elevated peroxisomal biogenesis and fatty acid oxidation (Figure 4B). Mitochondrion-related upregulated proteins included long-chain fatty acid CoA ligase 1 (ACSL1), optic atrophy 3 protein homolog (OPA3), and mitochondrial pyruvate carrier 1 (MPC1) (Figure 4C). In addition, several proteins important for oxidative phosphorylation, including cytochrome b5 type B (MT-CYB), cytochrome b-c1 complex subunit 6 (UQCRH), and NADH dehydrogenase [ubiquinone] 1 alpha subcomplex subunit 3 (NDUFA3) were upregulated following lanifibranor treatment (Figure 4D), confirming that pan-PPAR agonism enhanced processes related to peroxisomes and mitochondria. Most of the highest upregulated proteins related to lipid metabolism were also peroxisomal proteins (Figure 4E), further substantiating the efficacy of lanifibranor in triggering the expression of peroxisomal proteins. Notably, lanifibranor decreased the expression of multiple fibrosis-related extracellular matrix proteins, such as collagen alpha-2(I) chain (COL1A2) and collagen alpha-1(XIV) chain (COL14A1) (Figure 4F). Thus, lanifibranor upregulated proteins of lipid metabolism and downregulated proteins associated with fibrosis.

### Lanifibranor Treatment Has a Minor Impact on Liver Lipid Levels and Histology

**Figure 5:**
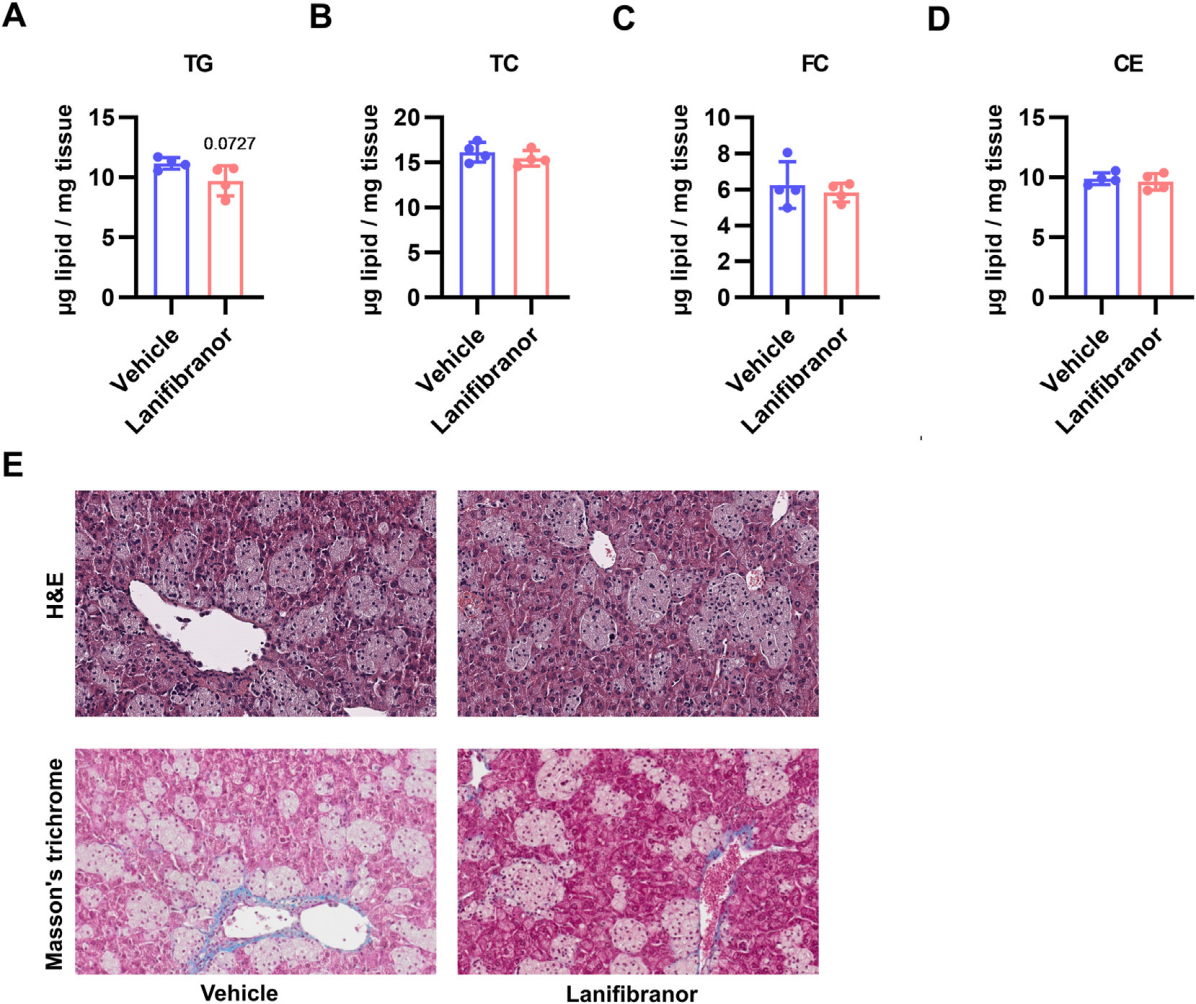
Unaltered liver lipid levels and histology after lanifibranor treatment of Lal−/− mice. Concentrations of hepatic (A) triacylglycerols (TG), (B) total cholesterol (TC), (C) free cholesterol (FC), and (D) cholesteryl esters (CE) in vehicle- and lanifibranor-treated Lal−/− mice. Data represent means ± SD (n = 4). Statistical significance was determined by 2-tailed Student's *t*-tests. (E) Liver hematoxylin and eosin (H&E) (top row) and Masson's trichrome staining (bottom row).

Despite the observed upregulation of 92 proteins related to lipid metabolism (Figure 4A), hepatic TG, total and free cholesterol as well as cholesteryl ester concentrations remained unaltered (Figure 5A–D). Liver histology was unchanged, as evidenced by hematoxylin and eosin staining (Figure 5E) and comparable hepatic collagen content (Figure 5E), suggesting that despite significant proteome changes, lanifibranor treatment had little effect on hepatic steatosis and pathohistology.

**Figure 6:**
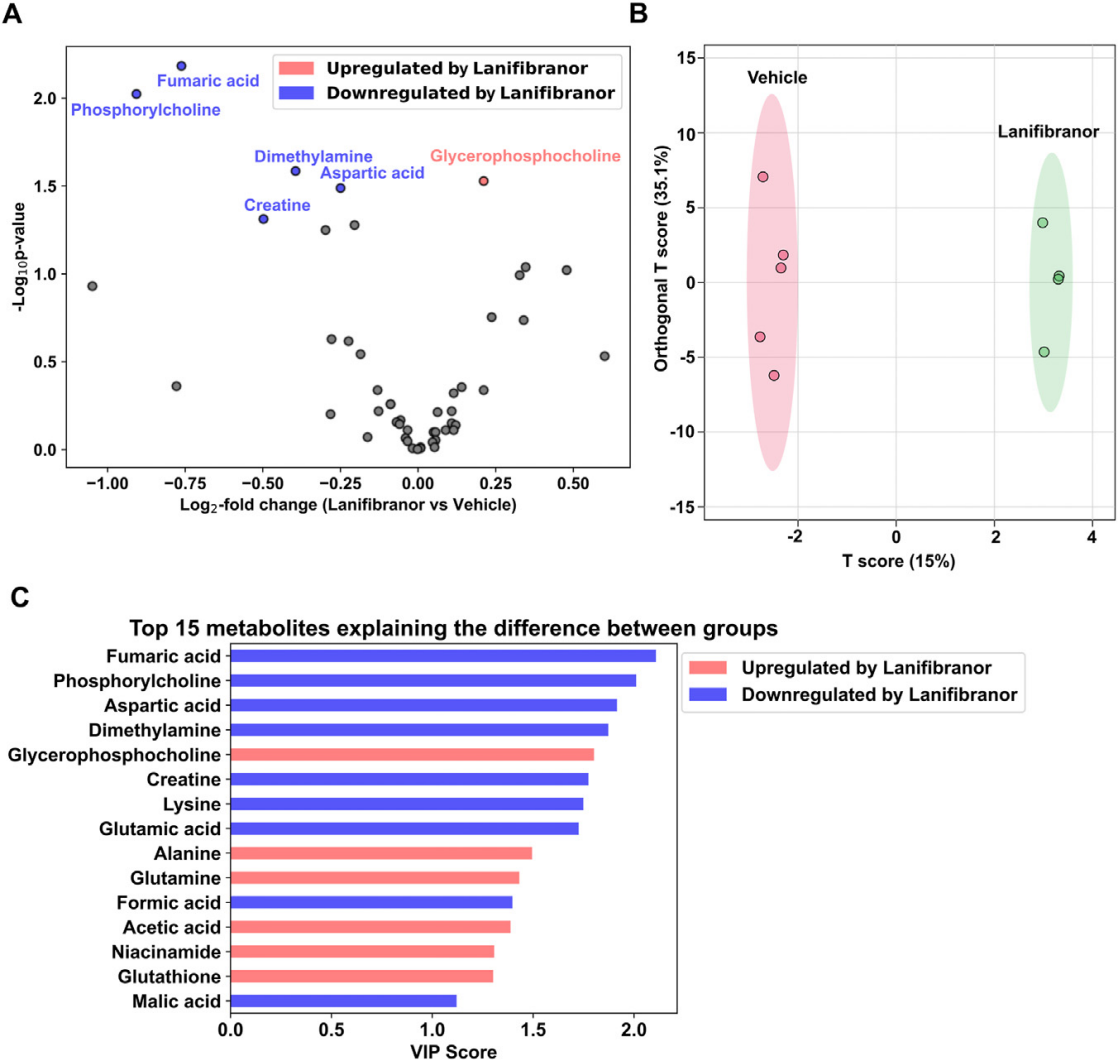
Treatment with lanifibranor leads to changes in liver metabolite concentrations in Lal−/− mice. (A) Volcano plot of metabolites with different abundances in the livers of vehicle- and lanifibranor-treated Lal−/− mice. (B) Orthogonal partial least squares discriminant analysis (O-PLS-DA) plot of liver metabolites and (C) ranking of metabolites based on the variable importance during projection (VIP) score. Data represent n = 4–5. (A) Statistical significance was determined by 2-tailed Student's *t*-tests with Benjamini-Hochberg correction (raw *P* value was plotted).

To gain further insight into the consequences of lanifibranor treatment on hepatic metabolism, we performed a targeted metabolomic analysis, which revealed obvious changes in the liver metabolome upon pan-PPAR agonist treatment (Figure 6A). To determine whether metabolites differed significantly between lanifibranor-treated and control livers and to identify the metabolites primarily contributing to this difference, we applied multivariate orthogonal partial least squares discriminant analysis. This revealed a clear distinction between the groups (Figure 6B). Metabolites with increased hepatic concentrations upon treatment (filtered by their high score in the variable of importance during projection analysis) included glycerophosphocholine, alanine, glutamine, acetic acid, niacinamide, and glutathione (Figure 6C). In contrast, fumaric acid, phosphorylcholine, aspartic acid, dimethylamine, creatine, lysine, glutamic acid, formic acid, and malic acid were among the metabolites that were decreased in lanifibranor-treated Lal−/− livers (Figure 6C). These data suggest that lanifibranor treatment markedly affects the metabolome in the liver of Lal−/− mice.

### Improved Inflammation and Dyslipidemia Following Lanifibranor Treatment

**Figure 7:**
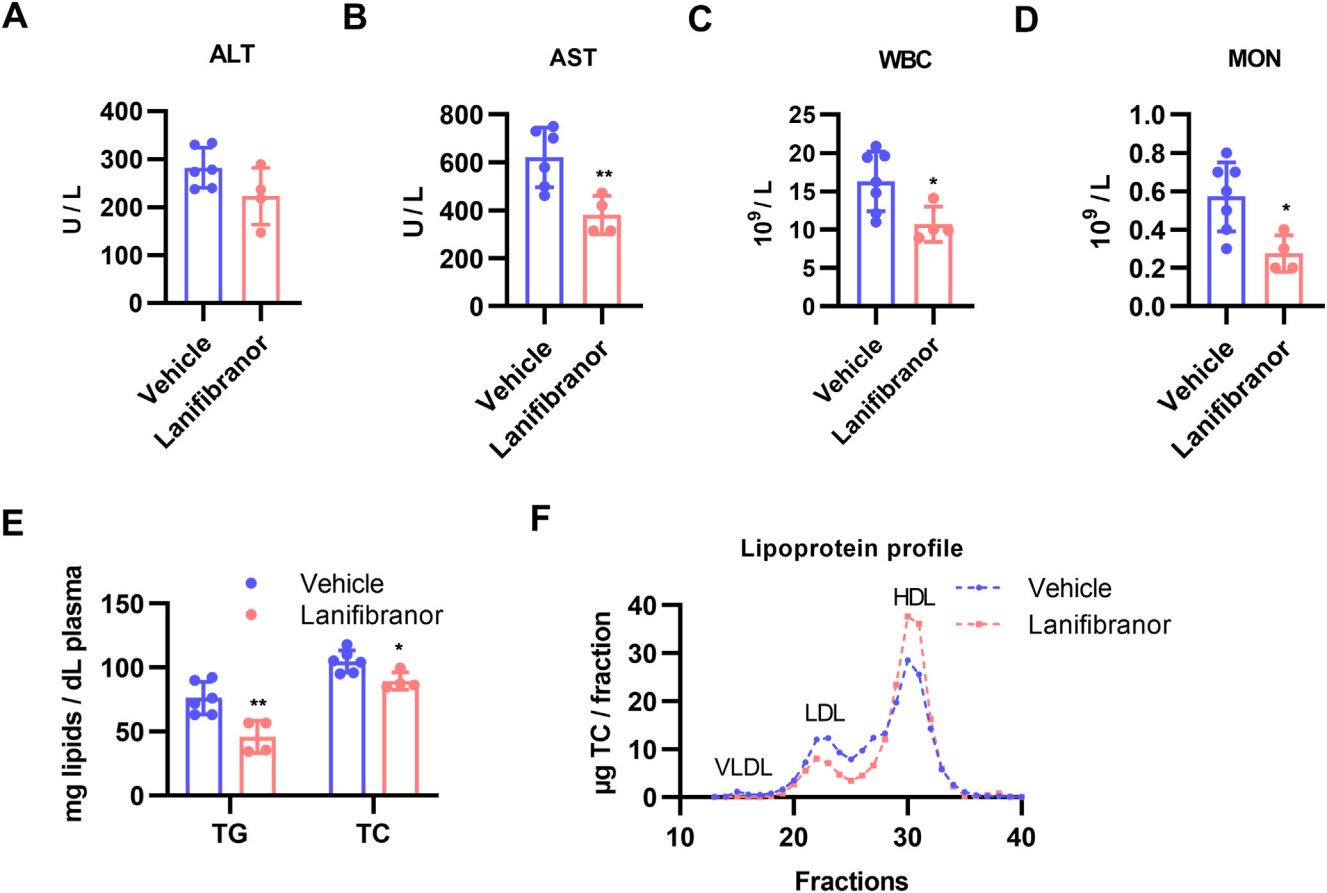
Lanifibranor treatment reduced liver inflammation and improved dyslipidemia in Lal−/− mice. Circulating levels of (A) alanine aminotransferase (ALT) and (B) aspartate aminotransferase (AST) in vehicle- and lanifibranor-treated Lal−/− mice. Number of (C) white blood cells (WBC) and (D) monocytes (MON), (E) plasma TG and TC concentrations, and (F) lipoprotein distribution in pooled samples from vehicle- and lanifibranor-treated Lal−/− mice. Data represent means ± SD (n = 4–6). ∗*P* < .05, ∗∗*P* ≤ .01. Statistical significance was determined by 2-tailed Student's *t*-tests.

Since the considerable changes in the proteome could not be explained by minor changes in liver lipid levels and histology, we tested whether treatment with the pan-PPAR agonist affected circulating lipid concentrations or systemic inflammation. We observed a trend toward lower plasma alanine aminotransferase (Figure 7A) and significantly decreased plasma AST levels (Figure 7B), indicating a reduction in hepatocellular damage. A marked reduction in circulating white blood cell (Figure 7C) and monocyte (Figure 7D) numbers suggested a beneficial impact of activated PPAR signaling on systemic inflammation. Finally, we examined the response of circulating lipid levels to lanifibranor treatment and observed decreased plasma TG and TC concentrations (Figure 7E) coupled with a reduction in low-density lipoprotein (LDL)-TC and an increase in high-density lipoprotein (HDL)-TC content. In contrast, blood glucose levels remained comparable (Figure A1D). These data indicate that lanifibranor attenuates liver and systemic inflammation while simultaneously improving dyslipidemia in Lal−/− mice.

## Discussion

Lanifibranor showed promising results in a phase 2b study in the treatment of patients with active MASH.^23^ Of the 247 patients enrolled in this study, 166 received either 1200 or 800 mg of lanifibranor once daily for 6 months and 81 patients were given placebo.^23^ The incidence of nausea, diarrhea, peripheral edema, anemia, and weight gain of approximately 3% occurred more frequently in the lanifibranor groups than in the placebo group. One patient developed mild heart failure, which was not attributable to the treatment. The results of this trial also indicated the potential for benefits with lanifibranor with respect to several secondary end points, including hepatic fibrosis, lipid profile, and glycemic control.^23^ An ongoing phase 3 clinical trial (NCT04849728) will further elucidate potential adverse effects. While lanifibranor has not yet been studied as a treatment option for LAL-D, it is plausible that it could offer therapeutic benefits, given the observed similarity in liver inflammation between patients with LAL-D and MASLD.^8^ The proteomic analysis confirmed that treatment with lanifibranor activated the PPAR signaling pathway in Lal−/− mice, with enriched terms related to fatty acid oxidation, peroxisomal biogenesis, and lipid metabolism. PEX11A and PEX7 were among the most upregulated peroxisome-related proteins and are essential for proper peroxisomal function and the prevention of dyslipidemia and obesity.^38^^,^^39^ The upregulated mitochondrial protein OPA3 is a crucial regulator of mitochondrial function,^40^ whereas the expression levels of ACSL1 and MPC1 are directly correlated with hepatic steatosis.^41^^,^^42^ However, counteracting the upregulation of proteins related to lipid metabolism, mitochondrial function, and fatty acid oxidation may have a superior effect, as these processes are impaired in Lal−/− compared to control mice.^17^ Following lanifibranor treatment, major components of oxidative phosphorylation were also upregulated, leading to a reduction in fumaric and malic acid, suggesting an increase in mitochondrial respiration. Consistent with several studies showing a favorable effect of pan-PPAR agonism on liver steatosis, inflammation, and fibrosis in mouse and rat models,^22^^,^^24^^,^^43^^,^^44^ lanifibranor decreased the expression of various fibrosis-related proteins in the livers of Lal−/− mice. In addition, the treatment increased the expression of glutathione, while reducing the concentration of circulating AST. This finding indicates that the reduced expression of proteins related to inflammation and fibrosis resulted in improved liver injury. Thus, despite the distinct pathologies of LAL-D and MASLD, lanifibranor has a beneficial effect on liver inflammation.

Lanifibranor treatment decreases hepatic TG concentrations in mice fed choline-deficient amino acid-defined high-fat diet (CDAA-HFD), high-fat/high-sucrose diet, and methionine-choline-deficient diet.^22^^,^^44^ In Lal−/− mice, lipids accumulate in lysosomes due to the absence of LAL.^8^ Consequently, these lipids cannot be mobilized, unlike lipids in cytosolic lipid droplets from livers affected by MASLD.^45^ Therefore, the reason why lanifibranor cannot alter TG and TC levels in the livers of Lal−/− mice may be due to the predominant accumulation of lipids within lysosomes and loss of a functional enzyme to degrade them upstream of the functional consequences of pan-PPAR agonism. Lipids accumulate in the livers of Lal−/− mice during the first weeks of life^46^ and starting lanifibranor treatment earlier may have a more pronounced impact on lipid metabolism and inflammation. For instance, feeding 5-week-old Lal−/− mice with a fenofibrate-enriched diet for 4 weeks reduced liver TC levels but did not affect TG concentration.^12^

Compared to the lanifibranor-promoted weight gain in MASH patients,^23^ the body weight of Lal−/− mice remained unchanged. However, lanifibranor treatment slightly increased the ratios of heart and liver to body weight in Lal−/− mice. In contrast, lanifibranor treatment reduced the liver-to-body weight ratio in WT mice fed a CDAA-HFD.^24^ However, it had no impact on body or liver weight in a rat model of liver cirrhosis^43^ or heart weight in rats^24^ and Alms1 mutant foz/foz mice. Heart weight was not discussed in other diet-induced models of MASLD.^44^ The phase 2b trial also showed no increase in heart-related complications in patients treated with lanifibranor.^23^ However, clinical trials have rarely assessed cardiovascular outcomes due to limited power in detecting the potential cardiovascular benefits of PPAR agonist treatment in MASLD patients.^47^ It would therefore be necessary to determine the influence of lanifibranor on heart weight and pathophysiology in various mouse models of MASLD. Despite an increased liver and heart weight relative to body weight in Lal−/− mice after lanifibranor treatment, spleen weight remained unchanged and no adverse effects on hematological parameters were observed. The slight increase in liver and heart weight in Lal−/− mice, without any changes in liver lipids and histology, might be attributed to the distinct pathophysiology present in LAL-D mice compared to genetic or diet-induced MASLD mouse and rat models. As lipid accumulation in LAL-D mice is already evident in the first few weeks of life,^46^ it is possible that young mice may exhibit signs of liver inflammation. Consequently, initiating treatment with lanifibranor at an earlier stage and/or a combination of lanifibranor with ERT may result in improved liver histology in LAL-D mice. Studies using lanifibranor in various animal models should also aim to assess organ weights to account for possible side effects of the treatment.

The decreased numbers of white blood cell and monocytes in lanifibranor-treated Lal−/− mice indicate decreased systemic inflammation, consistent with the known ability of PPAR signaling to modulate inflammation and macrophage infiltration.^48^ In addition, lanifibranor demonstrated a positive effect on plasma lipids by reducing TG and TC concentrations. This effect was also observed in control mice fed high-fat/high-sucrose diet, CDAA-HFD, western-type diet, methionine choline-deficient diet, in a mouse model of CCl_4_-induced liver fibrosis,^22^^,^^44^ as well as in MASH patients.^23^ To our knowledge, the consequences of lanifibranor treatment on plasma cholesterol levels in mouse models of MASLD or liver fibrosis have not been investigated. We also found an improved distribution of plasma lipoproteins following lanifibranor treatment, with decreased LDL-cholesterol and increased HDL-cholesterol levels. Similarly, HDL-cholesterol levels improved in lanifibranor-treated MASH patients, whereas LDL-cholesterol remained unchanged.^23^

In conclusion, the treatment with lanifibranor improved plasma levels of liver injury markers and lipid parameters, as well as lipoprotein distribution. Furthermore, it reduced the expression of various inflammation- and fibrosis-related proteins in the livers of Lal−/− mice. These findings suggest potential additive effects of lanifibranor to ERT that should be explored in future studies, as emphasized above.

## Supplementary Materials

Table A1
